# Is Suicide a Water Justice Issue? Investigating Long-Term Drinking Water Advisories and Suicide in First Nations in Canada

**DOI:** 10.3390/ijerph20054045

**Published:** 2023-02-24

**Authors:** Jeffrey Ansloos, Annelies Cooper

**Affiliations:** 1 School of Cities and Ontario Institute for Studies in Education, University of Toronto, Toronto, ON M5S 1V6, Canada; 2 Ontario Institute for Studies in Education, University of Toronto, Toronto, ON M5S 1V6, Canada

**Keywords:** suicide, mental health, Indigenous peoples, environment and health, water insecurity, long-term drinking water advisories, social determinants of health, ecopsychology

## Abstract

First Nations experience disproportionate rates of suicide when compared to the general population. Various risk factors are identified to increase understanding of the prevalence of suicide among First Nations, but environmental dimensions of suicide are understudied. This study asks whether water insecurity, as reflected by long-term drinking water advisories (LT-DWA), has any bearing on the distribution of suicide in First Nations across Canada, and specifically in Ontario. To assess this, we established the proportion of First Nations with LT-DWAs in Canada and in Ontario that have had suicides occur between 2011 and 2016 through a review of media archives. This proportion was compared to census data on the proportion of First Nations with suicides in Canada and in Ontario between 2011 and 2016, and statistical significance of difference was determined through chi-square goodness of fit test. Overall, the findings were mixed. Nationally, there was no significantly difference of proportion of First Nations with LT-DWAs with combined (confirmed and probable) reported suicides occurring when compared to census proportions; however, at the provincial level, findings had significant differences. The authors conclude that water insecurity in First Nations, as indicated by the presence of a LT-DWA in First Nations across may be an important environmental dimension of suicide, contributing to enhanced risk for suicide in First Nations.

## 1. Introduction

Suicide is one of the most urgent issues facing First Nations in Canada, with suicide rates approximately three times higher compared with the general population [1]. Global incident rates across a 60-year period suggest an upward trend of suicide among Indigenous peoples [2]. For First Nations in Canada, suicide is often cited as a leading cause of death for youth [3], and the leading cause of death for adults under the age of 44 [4]. Many risk factors are identified to increase understanding of the disproportionate prevalence of suicide among First Nations, but there is an urgent need to understand environmental dimensions. This exploratory study investigates one potential environmental dimension of suicide among First Nations: water insecurity. 

### 1.1. Suicide among First Nations in Canada

As early as 1995, the Royal Commission on Aboriginal Peoples categorized suicide risk factors for First Nations, including biopsychological factors (e.g., depression, substance use disorders), situational factors (e.g., disruptions to family life due to child welfare policies, off-reserve hospitalizations), socioeconomic factors (e.g., income levels, education levels), and cultural stressors (e.g., suppression of belief systems and spirituality, racial discrimination) [5]. Since then, a range of other demographic factors, including gender, age, and geographic location, have been found to have a significant bearing on rates of suicide among First Nations. Youth aged 15 to 24, men, and those living on reserve, are at higher risk for suicide [1]. Socioeconomic factors have also been consistently associated with high rates of suicide in First Nations [6,7,8]. Recently, household income, labour force status, education, and marital status accounted for 66% of suicide risk after adjusting for risk associated with age and sex among First Nations adults [1], demonstrating that socioeconomic factors have heavy bearing on suicide rates. However, to date, environmental dimensions of suicide among First Nations have received little research. 

### 1.2. Environmental Change and Water as an Environmental Health Issue in First Nations

Globally, Indigenous peoples face profound environmental changes, much of which is due to the steady growth of extractive industries [9,10]. These shifts are situated within a broader continuum of anthropocentric environmental change marked by 500 years of settler colonialism throughout the Americas [11]. Extractive industrial development is a feature of this continuum for which Canada is heavily invested, and First Nations heavily affected [12]. Mining, hydro-electric development, and oil and gas extraction are among some industries with noted health, social, cultural, and environmental impacts on First Nations in Canada [13,14,15,16,17,18,19,20,21,22,23,24,25,26,27]. 

Health inequity associated with these industries is enhanced by concurrent neglect by the Canadian government to uphold the rights of Indigenous peoples and ensure environmental regulation, which effects the “regulation for wastewater treatment, garbage disposal, hazardous waste, air pollution, and other environmental concerns” [28] (p. 99). Patterns of regulatory abandonment, in the service of extractive industry [29], coincides with the broader neglect of vital infrastructure in First Nations, such as those which provide access to clean water and support water security. Since 2015, the Canadian government has reported that there have been approximately 167 long-term drinking water advisories (LT-DWA), 31 of which remain in effect across 27 First Nations [30]. While most Canadians can access drinkable water, 27.5% of First Nations adults reported a lack of suitable drinking water; therefore, water insecurity is environmental racism [29,31,32,33,34,35]. With substantial numbers of long-term water advisories in effect in First Nations, such racialized forms of water insecurity warrant investigation as an environmental dimension of suicide. 

### 1.3. Theorizing Water Insecurity as an Environmental Dimensions of Suicide in First Nations

Against determinist models of public health, dimensional framing of environmental risk can be useful to help provide insight into the dynamic pathways and levels where risk for suicide is occurring in First Nations. Among these include proximal environmental dimensions, which refer to specific environmental factors, dynamics or processes that enhance suicide risk in ways that are immediately perceptible, and/or felt in the here and now. These might include safety threats to built and/or natural environments, inequities in environmental service accessibility (e.g., sewage treatment, water treatment), and threats to environmental security. 

Qualitative studies have suggested that First Nations affected by a lack of access to safe drinking water are likely to have disproportionately high suicide rates [28] and that water insecurity may be associated with mental health issues in First Nations [36], which by extension might intersect with suicide risk. Among Indigenous peoples in Canada, more broadly, it has been found that mental health outcomes associated with suicide may be affected by climate change, and in particular changes to land, ice, snow, weather, and sense of place [37,38,39]. Related to this, research has shown that environmental changes and related damages to environmental infrastructure accompanying climate change are linked to enhanced psychosocial distress, which in turn might affect suicidality [37]. In a recent study among Inuit in Nunatsiavut, climate change enhanced the likelihood of suicidal ideation [40,41]. 

Alternatively, distal environmental dimensions of suicide are those that describe factors that enhance suicide risk in ways that are more imperceptible, indirect, and/or functioning upstream. This includes matters such as disruptions to Indigenous people’s social and political self-determination through colonization. Colonization has come to be broadly recognized as a central determinant of Indigenous people’s health [42], as it produces pervasive social, political, economic, and environmental inequalities which have ripple effects on health outcomes [43], including on suicide in First Nations [44]. In addition to the political, social, economic, and environmental effects associated with colonization, assimilationist colonial policy and legal frameworks have also had negative impacts on cultural identity. The significance of this for understanding suicide risk cannot be understated, as cultural dislocation and identity confusion contribute to enhanced risk in First Nations, and given that continuity and a strong identity can be protective factors [45]. At the community level, the degree to which First Nations are engaged in actions that increase their direct control and self-governance over land has also been linked to lower suicide prevalence rates [45]. Such findings highlight how threats to Indigenous peoples’ sovereignty have health impacts that have bearing on suicidality [43,46]. However, distal factors also have bearing on matters of water governance. Indeed, as some qualitative studies have shown, colonial conceptions of water governance increase vulnerabilities to mental health issues and suicide among Indigenous peoples [47]. 

Taken together, environmental dimensions of suicide in First Nations likely occur across multiple levels. As a potential environmental dimension of suicide, water insecurity in First Nations is born out of a range of factors, including colonial water governance, and infrastructural violence [48,49]. Like the claims made about the relationship between water insecurity and suicide, it has also been suggested that the absence of adequate water treatment and sewage plants might affect rates of suicides among First Nations [29]. In this context the LT-DWAs might serve as a possible indicator of enhanced suicide risk.

### 1.4. Study Purpose 

The purpose of this study is to engage in exploratory analysis of water insecurity as an environmental dimension of suicide among First Nations, and specifically, water insecurity as represented by the long-term drinking water advisory. To assess this, we established what proportion of First Nations across Canada and Ontario with long-term drinking water advisories have had cases of suicide from 2011 to 2016, and determined if there is any statistically significant difference when compared with the national- and Ontario-specific proportion of First Nations that have had suicides enumerated in the last census within the same reference period [1]. 

## 2. Materials and Methods

To address the question of whether water insecurity (as reflected by the presence of a LT-DWA) might be an environmental dimension of health, we collected and analyzed data on First Nations with long-term water advisories in Canada and Ontario that have had suicides. Ontario was selected for a provincial level of analysis as it is a context that has received substantive media attention for having the most LT-DWAs among First Nations of any provincial jurisdiction in Canada. Ontario also has the largest number of First Nations of the provinces, with diverse geographical and socioeconomic representation. 

National- and Ontario-specific data were compared with proportions from a recent federal report on suicide among First Nations [1], which found that nationally, of the 555 of the First Nations enumerated in the 2011 National Household Survey by Statistics Canada, 40% had at least one incidence of suicide between 2011 and 2016. In Ontario, 29% of First Nations had at least one incidence of suicide between 2011 and 2016.

Given that the largely qualitative research base suggests that water insecurity might increase suicide risk, and that water infrastructure inaccessibility enhances risk associated with negative mental health outcomes, we expected to see statistically significant differences in the proportion of First Nations with long-term drinking water advisories that had suicides at both the provincial and national level. This design aimed to generate an exploratory and therefore preliminary examination of suicide in First Nations that combines both demographic and environmental variables, which in turn might inform First Nations suicide prevention, particularly at the level of future research and environmental policy. That said, this design is constrained by several variables, namely that actual suicide rates in each community are unknown and specific dates of suicide and LT-DWAs are not accounted for beyond the parameters of the stipulated reference periods. Therefore, interpretation of the analysis should be cautious, and future empirical studies should examine the issue with greater precision.

### 2.1. Materials

#### 2.1.1. First Nations Suicide Data

Our study explores the proportion of First Nations affected by long-term drinking water advisories (LT-DWAs) across Canada, and specifically in Ontario, that have had suicides between 2011–2016. To assess and compare the proportion of First Nations impacted by suicide, we cross referenced lists of First Nations with LT-DWA across Canada, available from Indigenous Services Canada [30], and First Nations in Ontario, available from the Ministry of Crown-Indigenous Relations and Northern Affairs Canada [50], with media reporting on suicides in First Nations. To do this, we performed a search for each First Nation to identify the existence of reported suicides through a Boolean search for “[name of First Nation]” AND “suicide” in the ProQuest Canadian Newsstream database, which compiles over 400 Canadian news sources from Canada’s leading media publishers. Publications from 1 January 2011 to 31 December 2016 were included. Google Advanced search was used to supplement this resource. With these searches, a First Nation was labelled “Y” for “Yes to confirmed reported suicide” if a source indicated the existence of at least one case of a reported death by suicide in the First Nation. Importantly, the data do not provide total annual counts for each First Nation, but rather whether at least one case of suicide was reported. 

During data collection, a lack of media reports on individual cases of deaths by suicide emerged alongside evidence of the existence of an issue of deaths by suicides in First Nations (e.g., quotes from a First Nation Chief identifying a suicide crisis or issue, the initiation of programs and/or resources to address deaths by suicide in the community, or grassroots-led initiatives to raise awareness of suicide and suicide prevention). These cases were categorized as “P” for “Probable suicides” when there was sufficient evidence to demonstrate community responses to deaths by suicide. The criteria for sufficient evidence included the existence of multiple reports for the same First Nation. In addition to the documenting of confirmed and probable cases, we also created a combined category.

#### 2.1.2. Long-Term Drinking Water Advisory (LT-DWA) Data

First Nations with LT-DWAs in Ontario and across Canada between 2011–2016 were identified through the open access “Status of Long-Term Drinking Water Advisories on Public Systems on Reserves” database compiled by Indigenous Services Canada [30]. The database documents LT-DWAs from the year 2000 to present. A LT-DWA is defined as a long-term drinking water advisory that has been in effect for longer than 12 months. 

### 2.2. Methods of Analysis

Drawing on the above data sources, two methods are used: First, frequency tables were created to show the proportion of First Nations across Canada, and specifically in Ontario with LT-DWA that had at least one or more suicides (confirmed, probable, and combined) between the years 2011 and 2016. Second, a chi-squared (X^2^) goodness of fit test was applied to all observations to determine whether differences of proportion between communities with LT-DWAs with suicides were significantly different from the proportion of First Nations with suicides as enumerated in the 2011 National Household Survey by Statistics Canada [1]. Analyses were conducted comparing a harmonized reference period for 2011-16. A chi-square (X^2^) goodness of fit test was appropriate because it evaluates whether proportions of categorical or discrete outcomes follow or differ significantly from population distributions. Importantly, for our comparative analysis, population distribution does not refer to suicide rates, but to the proportion of communities that had suicides occur in them, at least once between 2011 and2016. In such an analysis, the null hypothesis would be that our suicide data follows the distribution of currently reported proportions, and the alternative hypothesis is that it does not follow the same distribution. In this study, we set our significance level of *p* ≤ 0.05. Within such an analysis, significant differences must be interpreted cautiously, as differences could hypothetically be accounted for by a range of other variables. For this exploratory study, significant differences point towards the potential of LT-DWA to enhance suicide risk, but findings should be read as tentative. Consideration of water insecurity as an environmental dimension of suicide in First Nations must account for other variables (i.e., time, suicide rates) in design and therefore, broader investigation beyond this study is needed. 

### 2.3. Ethics 

Given that our research involves the secondary use of publicly assessable data, which is provided without any identifiers which would allow attribution of private information to an individual, our study is exempt from institutional ethics review. However, given the sensitive nature of the issue, and the size of First Nations communities, we do not present any linked identification of First Nations affected by suicide. This is aligned with the principles of “respect for persons”, “justice”, and “concern for welfare”, key to ethical research involving First Nations people in Canada [51] (p. 110). At the same time, we acknowledge that this information already exists in the public domain, though not in an aggregated manner. To ensure cultural safety in access and appropriate use, members of our research team included citizens of First Nations (including the first author). While ownership, control, access, and possession of the disaggregate data is public, aligned with principles of Indigenous data sovereignty, access to the aggregated dataset used in this study is freely available to First Nations upon request. 

## 3. Findings

### 3.1. First Nations with Long Term Drinking Water Advisories in Canada 

Frequencies for First Nations with LT-DWAs with and without suicide across Canada compared to national reporting of the proportion of First Nations across Canada with and without suicide between 2011 and 2016 are shown in Table 1.

As shown above, when analyzing the combined reports of confirmed and probable suicides, no significant difference was noted. As such, the null hypothesis is accepted. While significant differences exist in isolation for confirmed and probable suicides––in both cases, less than the census reporting of national proportions––overall, the combined figure is likely a closer approximation of actual cases. 

### 3.2. First Nations with Long Term Drinking Water Advisories in Ontario

Frequencies for First Nations with LT-DWAs with and without suicide in Ontario compared to the reported provincial proportion of First Nations with and without suicide between 2011 and 2016 are shown in Table 2. Please note that the frequency distributions for Ontario First Nations without LT-DWAs are also shown, as these figures are currently enumerated in the available data, unlike the national distributions highlighted in Table 1. 

As shown above, the proportion of First Nations in Ontario with LT-DWAs with suicides (combined confirmed and probable) within the 2011–2016 reference period is significantly higher in comparison with Statistic Canada reported figure of 29% [1]. As such, the null hypothesis is rejected, and the alternative hypothesis accepted. Additionally, it is notable that compared with the proportions of those communities without LT-DWAs, First Nations with LT-DWAs were more likely to have reported suicides between 2011–2016.

## 4. Discussion

### Environmental Dimensions of Suicide in First Nations

The results of this study signal the importance of water insecurity, as indicated by the presence of a LT-DWA in First Nations, as a likely environmental dimension of suicide. The provincial findings from Ontario make a particularly strong case for understanding LT-DWAs in this way. Given that both the proportions of First Nations with LT-DWAs in Ontario were significantly higher than Ontario census data, as well as experiencing a higher proportion of suicide than First Nations without LT-DWAs, overall this lends support to the theory of increased of risk of suicide associated with LT-DWAs. Our findings at the national level are less compelling. The lack of significant difference in proportions compared to census data at the national level coupled with the lack of enumeration of suicides within First Nations at the national level without LT-DWAs, constrains what can be said about this theory at the national level. Nonetheless, given that Ontario represents the largest concentration of First Nations under LT-DWAs among the Canadian provincial and territorial jurisdictions, our study findings point to the vital importance of further investigation of the impact of LT-DWA on suicide in First Nations. The extent to which distribution might be affected by other variables not accounted for in this analysis is an important consideration, and caution should be used when interpreting the significance of these findings. Furthermore, given the potential for underreporting, particularly in media contexts, our findings maybe under representative. 

What does seem to clear is that while LT-DWAs have long been seen as infrastructural indicator of environmental racism, they are a relevant dimension of suicide among First Nations in Ontario. They are certainly relevant, the extent to which remains to be seen nationally. Research has highlighted that “the extremely high numbers of First Nations who have unsafe drinking water and are under boil water advisories… places First Nations at increased risk of death” [52] (p. 115). When compared with communities not facing similar water issues, our study makes clear that suicide can follow such environmental and infrastructural conditions.

Our findings also resonate with qualitative studies that have suggested potential links between environmental dimensions and suicide in First Nations. Specifically, in places where access to safe drinking water is made vulnerable––due to failed infrastructure, environmental racism, and environmental changes––heightened psychosocial stress, mental health concerns, and suicidality may be more likely to occur [28,36,37]. This does not denote a causal or deterministic relationship, but rather, that LT-DWAs are a part of a vast constellation of relevant factors.

Conceptually, some research has theorized that suicide in Indigenous community contexts might be understood as an embodied expression or affective manifestation of the debilitating weight of settler-colonial, biopolitical, and necropolitical violence [46,53]. Our study helps to expand this assertion by pointing to the ways that suicide, at least in how it is distributed in Ontario, is linked to infrastructural issues and environmental policy. Additionally, while our study does not directly assess the affective mechanisms of this relationship, the findings do provide further grounds for exploratory investigation into whether the risk for suicide is enhanced by water insecurity. Therefore, theories of suicide must also account for the biosociality of infrastructural and environmental issues. This is generally consistent with research highlighting that mental health outcomes associated with suicide and suicidality in Indigenous communities are likely shaped by environmental changes [37,38,39,40,41]. Indeed, all contexts of LT-DWAs are also ecologies of environmental change. 

Most critically, this exploratory study holds what Indigenous peoples have long indicated about the relationship between environmental health, and affective and embodied dimensions of health as critically important for ongoing and future suicide research: that “violence on the land is violence on the body” [26] (p. 1). Colonialism is materially felt and manifest in the relationship between water insecurity and suicide, and our study, while constrained by factors including limited data and mixed national findings, signals the importance of material analyses of suicidal risk. 

While a LT-DWA can be framed as a public health notice concerning water quality, the racialized distribution of water insecurity elucidates the ways that the LT-DWA can come to be seen as emblematic of broader forms of structural violence. Put another way, LT-DWA can be understood as indicative of a particular political predicament facing First Nations. Access to clean drinkable water is undermined by the Federal government’s administration in a manner that would be intolerable for the broader public but that operate as a state of exception in First Nations [31]. It is notable that threats to self-determination are widely regarded as a risk factor for suicide in First Nations [45], and resonantly, our findings further justification to implicate a wider array of political processes that have bearing on suicidal risk, and certainly within research. As some research has suggested, suicide prevention in First Nations is a domain where questions of justice are negotiated [44,46,54], and in this case, questions over Indigenous people’s water sovereignty take center stage. 

Relatedly, this study also raises important questions about the field of suicide prevention, particularly strategies that extend beyond domains typically associated with prevention (e.g., mental health, social services, public safety, educational services). Suicide prevention is often addressed downstream, and with limited efficacy at the level of direct services, crisis intervention, and risk assessment. Conversely, our study in trying to explore the risk posed by water insecurity, raises the possibility that water security is an important upstream avenue for suicide prevention. While all First Nations deserve water security, and it suicide crisis should not be seen as a requisite pre-condition to achieve such ends, many First Nations in Canada are languishing under governmental inaction. If water insecurity is a key dimension enhancing risk for suicide in First Nations, then environmental policy related to water security, be it in infrastructure or governance, is a vital dimension that must be considered and evaluated. 

Addressing such dimensions of health is not without challenge and requires a degree of political engagement and responsivity for which the Canadian government has a deleterious record. The findings of this study may help to advance existing and ongoing environmental justice organizing and advocacy by and on behalf of Indigenous peoples within several realms: legal advocacy; the restructuring of environmental impact assessments and regulatory frameworks; and Indigenous movements to reimagine water governance, including grassroots initiatives that place water governance under Indigenous authority and jurisdiction. 

Much can be learned from First Nations that have already begun turning to legal means to pursue justice for damages felt by communities because of long-term water insecurity. A class action lawsuit led by Tataskweyak Cree Nation, Neskantaga First Nation, and Curve Lake First Nation for damages related to LT-DWAs resulted in a historic settlement agreement that recognized Canada’s failure to take all reasonable steps to provide First Nations with safe drinking water. The affidavits of community members attested to the impact of the lack of safe water on the community on mental health and suicide in the communities [55]. The settlement agreement recognizes these mental health impacts of water insecurity on First Nations and provides compensation to all First Nation individuals who have lived under a water advisory for at least one year between 1995 and 2021. Individuals can apply for additional compensation for injuries including demonstrated impacts to mental health [56]. However, there is further advocacy that can be taken up in this area regarding the systemic impacts that water insecurity may have on mental health and suicidality in First Nation communities. This study provides an important evidentiary finding to support First Nations legal pursuits to redress systemic impacts of water insecurity, including the potential of enhanced risk for suicide by LT-DWAs. At an international scale, it is also noteworthy that the reduction and eventual elimination of deaths by suicide is an indicator of progress on the United Nations Sustainable Development Goal (SDG) 3 Good Health and Wellbeing with mechanisms for observation around the world [57]. Beyond the primary methods of engagement (e.g., youth socioemotional literacy, prevention, and mental health service equity), Indigenous environmental governance and water security might be considered for inclusion in population-based strategies for achieving this SDG. Similarly, the United Nations has a Special Rapporteur mechanism for monitoring and implementation of Indigenous Peoples Rights. While concerns about water insecurity among Indigenous peoples in Canada have been highlighted by the Special Rapporteur [9], ongoing monitoring should also take into consideration the potential impacts of racialized water insecurity on mental health and Indigenous life expectancy in Canada. 

The lens of water insecurity as an environmental dimension of suicide requires greater scrutiny over impacts to water systems when prospective projects on First Nations territories are proposed by governments and industry. Environmental impact assessments (EIA) should consider the distal and proximal effects on mental health and suicide. While some EIAs consider social, cultural and health impacts, and calls for pairing EIAs with standardized Health Impact Assessments (HIAs) hold promise [58,59], consideration of mental health and suicide is a persistent gap that needs urgent attention in environmental policy. With some exceptions [60,61,62] mental health and suicide are almost entirely unexamined with regard to impact assessment among Indigenous communities in the North American context. Moreover, when these impacts are considered and/or identified, the responses proposed by government and industry are almost always an emphasis on strengthening access to health services, instead of increased regulation, demonstrating a failure to act on key sources of distress and underlying causes [58]. Demonstrating that water-related environmental impacts might have critical effects on mental health and suicide can advance First Nation advocacy for better mitigation of impacts to water systems and water-related infrastructure and community suicide prevention initiatives.

However, for suicide as a distinct impact of environmental disturbances to be effectively integrated into EIAs/HIAs, in addition to further empirical research, it is critical to consider who has the authority in structuring an assessment process and whether Indigenous communities have meaningful decision-making authority. Indigenous water protectors and environmental advocates have argued that more is needed than participation through the “optics of consultation” [63] (p. 4) provided by impact assessments processes. Conventional impact assessments fail to fundamentally shift decision-making authority and governance of water and water systems. Such processes are shown to have limited impact on the ability of Indigenous communities to influence their outcomes [64,65] unless meaningful co-management takes place throughout all phases of the process [66] and have Indigenous leadership [67,68,69]. Our findings also hold implications for thinking beyond project-based impact assessment to a wholesale shift towards Indigenous water governance, which would dismantle colonial frameworks and regulatory structures and empower Indigenous self-determination over water management [63,70,71,72].

The Treaty #3 Nibi (Water) Declaration is an important example which explicitly links Indigenous governance of water to mental and emotional health, as well as livability more broadly [63,73]. As an approach to suicide prevention, self-determined water governance is also resonant with long-standing evidence on suicide prevention among First Nations. Grouped under the category of cultural continuity, Indigenous self-determination over matters of governance, be it in health, social, educational, public safety, and land claims processes, are protective factors [45]. Our findings point towards the significance of water and environmental governance sovereignty as another cultural continuity factor which might have a protective bearing on suicide.

## 5. Limitations and Directions for Future Research

There are several limitations to this study and implications for future directions in research. This study is constrained by several challenges in terms of design, and relatedly by the (un)availability and (in)accessibility of relevant data.

Beginning with design, we wish to reiterate that to establish a strong empirical foundation for role of LT-DWAs in enhancing suicide risk, more advanced and rigorous design is needed, along with confirmatory analysis of our findings. Ideally, the most effective way to study this would involve comparison of First Nation suicide rates without LT-DWAs to First Nation suicide rates with LT-DWAs across time, while seeking to control for and normalize other relevant variables. Our dataset simply does not provide this level of detail or precision. Vital statistical data related to suicide is heavily restricted in the Canadian context, even for qualified researchers, and this is especially true regarding suicide data specific to First Nations. Access and specificity of data is complicated by inconsistent standards across Canadian jurisdictions for tracking race-based data of suicide cases limiting knowledge of population occurrence in specific groups, including First Nations. Even at the census level, most data related to suicide deal with probabilistic datasets, meaning little is known about actual case rates in First Nations.

Our formulation of an archival database of media reports faces similar constraints. While mainstream media reporting on suicide is generally considered a reliable source, its use as suicide data in our study is likely subject to some error: First, it is possible, particularly in our assessment of probable cases, that there may be some false-negative and false-positive cases based on the available evidence. Typically, people do not falsely claim if a suicide has occurred, and/or if suicide is a pressing local issue. We took media reporting in First Nations at face value. Second, due to privacy reasons, it is assumed that media coverage does not cover all cases of suicide in First Nations, and therefore, as a source, it may have missing data, that we have simply coded as non-cases. While a limitation, this is true of all data sources on suicide. For example, the use of census and coronal data related to suicide may underreport due to the “difficult nature of classifying suicide and the time delay in determining this as the cause of death, which may vary from year to year and one region to another” [74] (p. 1). Additionally, other factors include that the cause of death is often treated as confidential by many families and communities. It has been suggested that recent population level suicide rates for First Nations may underestimate as well [1]. For Ontario numbers, this is exacerbated by issues of coroners failing to attend deaths in Northern Ontario First Nations communities in the event of fire deaths and suicides [75]. Additionally, much of the information on suicide rates is on the regional health authority level, not the level of First Nations.

Decisions on scoping the data collection, such as a provincial analysis of Ontario, but not other provinces, was shaped by the constraints of our current data, although future investigations may examine other regions as well as augment the national analysis. Further, the focus on First Nations and on-reserve deaths adds limitations. The exclusive focus on First Nations means this study is unable to speak to or address the impact of LT-DWAs and suicide occurring among Métis and Inuit, as well as urban, off-reserve, and non-status Indigenous peoples. Additionally, given the focus on First Nations as the population, little is known about the ways water insecurity might be shaping Indigenous people’s experiences with suicide off-reserve. By including only deaths on reserves, we are conscious of under reporting of the impacts of suicides that happen away from home. For example, in our data collection around northern Ontario communities, a known issue is the suicides of youth from Northern Ontario First Nations who are attending school in southern communities such as Thunder Bay and Sioux Lookout [76]. 

An additional limitation is that while we investigated water insecurity in reference to the presence of a LT-DWA, a parallel dimension which we did not explore is the effect of water security, and the impact of interventions to enhance water security. This was in part shaped by limitations of our data. While the absence of a LT-DWA was taken up in our provincial analysis, this does not necessarily equate to water security. Future research should examine this factor, including differences in suicide distributions between First Nations with and without LT-DWAs, and whether the prevalence of suicide decreases in communities where LT-DWAs have been lifted.

Our study points towards the need for increased research on data governance related to suicide in First Nations. It is notable that the Truth and Reconciliation Commission Call to Action 55 explicitly calls for annual reporting on health outcomes and suicide data within Indigenous communities [77]. Nonetheless, under the present approach to suicide data governance, such reporting is absent and/or disparate. Research is urgently needed to address such barriers. Facing such constraints, First Nations can also pursue self-governing vital statistics. For example, Nishnawbe Aski Nation has documented deaths by suicide since 1986 across their 49 member communities, providing them with the largest database of cases in First Nations. Such an approach would allow for much more effective suicide prevention research and supports the goals of Indigenous data sovereignty.

Our study also raises several important questions of how we measure environmental impacts on issues like suicide. Given the methodological challenges associated with suicide research, in addition to epidemiological research, a significant emphasis has been placed on psychological autopsy as a method of inquiry in the wake of a suicide. Our study gestures towards conceptualization of autopsy at a structural and/or environmental level. Rather than attending to a specific incident, attending to structural and/or environmental analysis at the level of a First Nation might provide more contextual information about distal and proximal dimensions of environmental risk. Structural and/or environmental autopsy might provide to be a promising direction for suicide research. At the same time, prevention research ought to also engage more fulsomely with people with lived experience of suicide. This might include people with direct experience of suicidality, as well as those supporting or affected by a suicide. Our study strengthens a rationale for the need for in-depth qualitative and community-based research examining environmental dimensions.

Finally, our study also raises important questions about additional indicators of environmental dimensions of suicide. There may be other ways of assessing the impact of water insecurity that deserve further attention, such as indicators related to waste water management and housing infrastructure for drinking water. More broadly, given the intersection of extractive industries with other demonstrated health, social, and environmental impacts, it is vital that the connection to suicide be more fully examined.

## 6. Conclusions

In summary, this article has highlighted the importance of water justice as potential environmental dimension of preventing suicide in First Nations. While substantial gaps remain in our knowledge on the environmental dimensions of suicide in First Nations, and Indigenous communities more broadly, this study makes an important contribution highlighting how water insecurity can contribute to enhanced risk. The extent to which it enhances risk needs further study. While the affective mechanisms of the relationship between suicide and environmental dimensions remain out of sight, what is clear is that water insecurity and suicide are matters of justice.

Prevention efforts must address issues of environmental racism and infrastructural violence. While frontline service delivery for mental health is an important facet of suicide prevention, from an upstream perspective, remediating the impacts of sustained environmental racism and infrastructural violence is a critical priority that should shape prevention strategy. In practice, this means that we must work to rapidly end water insecurity in Indigenous communities, and address the impacts of colonial water governance, pollution, and other sources of water insecurity. Together with First Nations, we must also work towards prevention at the level of enhanced self-determination in environmental management, promoting environmental impact assessment processes that account for mental health impacts, enhancing environmental regulation, and Indigenous-led environmental policy in water governance.

Suicide prevention is a water justice issue because water is life. Against the violence of colonialism, the protection of water is the protection of life. Conceptually, it follows that suicide might be prevented when we nourish conditions for living. In other words, we promote life by ensuring water justice.

## Figures and Tables

**Table 1:** Suicide Distributions for First Nations with Long Term Drinking Water Advisories (LT-DWAs) Across Canada Compared to Reported National Proportion of First Nations with Suicide Between 2011 and 2016.

First Nations with Long Term Drinking Water Advisories in Canada between 2011 and 2016 (N = 47)	n	%	χ ^2^ *	*p* -Value *
confirmed suicides	10	21%	15.04	0.00
probable suicides	8	17%	22.04	0.00
confirmed and probable suicides	18	38%	0.17	0.68
no reported suicides	29	62%	0.17	0.68

* Proportion of First Nations with LT-DWA compared to the national proportion of 40% First Nations with suicide reported by Statistics Canada [1], except for final row calculated against 60% that do not have suicides.

**Table 2:** Suicide Distributions for First Nations with and without Long Term Drinking Water Advisories (LT-DWAs) in Ontario Compared to Reported Provincial Proportion of First Nations with Suicides Between 2011–2016.

First Nations in Ontario (N = 140)	Reported Suicides	n	%	χ ^2^ *	*p* -Value *
With LT-DWA (n = 18)					
	Confirmed	6	33%	0.78	0.38
	Probable	3	17%	6.99	0.00
	Combined confirmed and probable	9	50%	21.42	0.00
	None	9	50%	21.42	0.00
Without LT-DWA (n = 122)					
	Confirmed	13	11%	15.73	0.00
	Probable	5	3%	32.83	1.00
	Combined confirmed and probable	18	15%	9.52	0.00
	None	104	85%	9.52	0.00

* Proportion of First Nations with LT-DWA compared to the provincial proportion of 29% of First Nations with suicide reported by Statistics Canada [1], except for final row calculated against 71% that do not have suicides.

## Data Availability

All data presented in the study are publicly available in disaggregate form. The aggregated dataset used in this study is available for use on request from the corresponding author. Data related to Long-Term Drinking Water Advisories in First Nations in Ontario and across Canada was obtained from the “Status of Long-Term Drinking Water Advisories on Public Systems on Reserves” database and is available open access from Indigenous Services Canada at https://www.sac-isc.gc.ca/eng/1620925418298/1620925434679 (accessed on 15 September 2022). Data related to suicides in First Nations in Canada and Ontario was obtained through a review of media archives through the ProQuest Canadian Newsstream database, which compiles over 400 Canadian news sources from Canada’s leading media publishers. While not a public archive, hyperlinks to media archive records are available on request from the corresponding author.
